# In Vitro Assessment of Antiviral Activity of Saikosaponin B2 Against Porcine Epidemic Diarrheal Virus

**DOI:** 10.3390/v18090997

**Published:** 2026-09-10

**Authors:** Han Zhang, Yue Liu, Fan Zhang, Yibo Xu, Wanli Sha, Shushuai Yi, Baishuang Yin

**Affiliations:** 1 College of Animal Science and Technology, Jilin Agricultural Science and Technology College, Jilin 132101, China; zhanghan001225@163.com (H.Z.); shawanli@jlnku.edu.cn (W.S.); 2 College of Animal Science and Technology, Jilin Agricultural University, Changchun 130118, China; 13321442582@163.com (F.Z.);; 3 Jilin Province Cross Regional Cooperation Technology Innovation Center of Porcine Main Disease Prevention and Control, Jilin 132101, China

**Keywords:** PEDV, saikosaponin B2, natural herbal monomer, antiviral activity, variant strain

## Abstract

Porcine epidemic diarrhea virus (PEDV) variant strains lead to vaccine failures and significant economic losses in swine farming, highlighting the need for new natural small-molecule antiviral candidates. In this study, we screened a library of 45 small molecules using a cytopathic effect (CPE)-based cytoprotective assay. Saikosaponin B2 (SSB2), a triterpenoid saponin derived from Bupleuri Radix, emerged as the most promising candidate, boasting a selectivity index of 19.77, which surpasses that of most natural compounds. Dose–response tests confirmed SSB2’s concentration-dependent anti-PEDV activity, with an inhibitory plateau reached at concentrations above 60 μmol/L (μM). The 50 half-maximal effective concentration (EC_50_) values differed between CPE reduction and viral titer readouts, a discrepancy commonly due to varying detection sensitivities. Mechanistic assays revealed that SSB2 does not possess direct virucidal properties; instead, its antiviral efficacy depends on sustained treatment to inhibit viral adsorption and internalization. Time-of-addition experiments further demonstrated that SSB2 effectively suppresses both early viral entry and mid-stage intracellular genome replication, without affecting viral release. Notably, post-infection treatment resulted in stronger inhibition compared to continuous administration, possibly due to disrupted cellular proliferation homeostasis from prolonged exposure. Additionally, SSB2 maintained strong inhibitory effects against prevalent G2a (JL-06) and G2b (JL-08) field variants, though its efficacy was reduced for both strains and slightly weaker against JL-08 than JL-06. Overall, this study systematically characterizes SSB2’s dual-stage anti-PEDV profile and its broad-spectrum activity against spike-mutant strains, supporting SSB2 as a promising herbal lead for developing veterinary antivirals targeting variant PEDV.

## 1. Introduction

Porcine epidemic diarrhea (PED), a devastating enteric disease caused by porcine epidemic diarrhea (PEDV), poses a severe threat to global swine production [1]. As a highly contagious pathogen, PEDV primarily infects neonatal piglets, triggering clinical signs including vomiting, acute watery diarrhea and severe dehydration. Without effective prevention and control strategies, PED outbreaks result in extremely high mortality in suckling piglets and cause enormous economic losses to the pig industry. Mutant PEDV variants began to circulate extensively across China in 2010, and large-scale regional epidemics subsequently emerged in North America and East Asia in 2013 [2,3].

Continuous amino acid substitutions within the spike (S) glycoprotein drive the evolutionary divergence of PEDV and modulate viral infectivity. PEDV isolates are classified into two principal genotypes, G1 and G2, with G2 subtypes currently prevailing in global field epidemics [4]. Conventional vaccines formulated based on early G1 strains can only partially mitigate epidemic risks; however, the newly prevalent G2 variants have greatly weakened vaccine-induced protective immunity [5,6]. Vaccination as a single prevention strategy fails to completely block PEDV transmission, highlighting the urgent need for complementary antiviral therapeutic agents.

Small-molecule antiviral agents serve as vital adjuncts to PEDV immunoprophylaxis. Systematic compound screening pipelines have been well established in severe acute respiratory syndrome coronavirus 2 (SARS-CoV-2) research, providing standard technical references for the discovery of anti-PEDV candidates [7,8]. Over the past decades, numerous synthetic antiviral molecules have been clinically applied, and their design frameworks have informed the development of anti-PEDV drugs [9,10,11]. Synthetic compounds are characterized by specific binding to viral targets and potent, rapid antiviral activity, whereas natural small-molecule monomers derived from medicinal herbs represent promising alternative lead compounds with unique strengths [12]. Phytochemical monomers generally exhibit low cytotoxicity, low propensity to induce viral drug resistance, and the advantage of being readily available as low-cost starting materials. These merits compensate for the common defects of synthetic antivirals, including tissue residue and high cytotoxicity at therapeutic concentrations [13]. Therefore, we constructed a compound library containing multiple validated synthetic and natural antiviral substances for parallel comparative screening in this study [14].

Among the diverse antiviral phytometabolites, pentacyclic triterpenoid saponins have been recognized as promising candidates against coronaviruses through systematic investigations [15]. *Bupleurum chinense* is a traditional Chinese medicinal herb, and saikosaponin B2 (SSB2), its major bioactive triterpenoid monomer, regulates host signaling cascades that govern cell proliferation and cellular microenvironment homeostasis [16,17]. Accumulating in vitro evidence confirms the broad-spectrum antiviral capacity of SSB2. Within safe concentration ranges, SSB2 blocks the early attachment and internalization of feline herpesvirus 1 [18], suppresses genomic replication of hepatitis C virus [19], and produces notable inhibitory effects on SARS-CoV-2 [20]. Other saikosaponin analogs also display suppressive activity against influenza A virus and measles virus [21]. However, no studies have investigated the anti-PEDV activity of SSB2, particularly against the prevalent G2 subtype strains. This research gap limits the translational development of SSB2 as a lead compound for the prevention and treatment of PEDV infections.

In this study, our results demonstrated that SSB2 screened from 45 small molecules exerted robust in vitro anti-PEDV activity. These findings provide theoretical support for the application of SSB2 in the treatment of PEDV infections.

## 2. Materials and Methods

### 2.1. Cells, Viruses, Compounds, and Reagents

Vero E6 cells (Catalog No. SCSP-5255, Cell Bank and Stem Cell Bank, Chinese Academy of Sciences) were cultured in Dulbecco’s Modified Eagle Medium (DMEM; Gibco, Monmouth Junction, NJ, USA) supplemented with 10% fetal bovine serum (FBS; VivaCell, Shanghai, China) and 1% penicillin–streptomycin–amphotericin B solution (Beyotime, Shanghai, China), and incubated at 37 °C under 5% CO_2_. PEDV strains CV777, JL-06/2023 (G2a subtype), and JL-08/2023 (G2b subtype) were isolated and identified in our laboratory. The titers of the stock viruses were 10^7.5^ TCID_50_/mL for CV777, 10^7.0^ TCID_50_/mL for JL-06/2023, and 10^6.5^ TCID_50_/mL for JL-08/2023. All small-molecule compounds and CCK-8 cell viability assay kit were purchased from MedChemExpress (MCE, Monmouth Junction, NJ, USA). Mouse monoclonal antibody targeting PEDV nucleocapsid (N) protein was obtained from Immunology Consultants Laboratory (ICL, Portland, OR, USA). FITC-conjugated goat anti-mouse IgG was supplied by Beyotime (Shanghai, China), HRP-conjugated goat anti-mouse IgG was obtained from Boster (Wuhan, China), and β-actin (D6A8) reference antibody was purchased from Cell Signaling Technology (CST, Danvers, MA, USA). A library of forty-five small-molecule compounds was specifically curated for initial antiviral screening. The selection criteria prioritized compounds with proven antiviral activity against coronaviruses or enteric RNA viruses from previous research. All compounds employed in this study were commercially available.

### 2.2. Cytotoxicity Assay

Vero E6 cells were seeded in 96-well plates at a density of 1 × 10^4^ cells/well and cultured for 12 h to allow complete attachment. After attachment, the medium was replaced with DMEM containing 2% FBS to maintain basal cell viability. All test compounds were dissolved in dimethyl sulfoxide (DMSO; MCE, Monmouth Junction, NJ, USA) and serially diluted two-fold in 2% FBS-DMEM to yield final concentrations ranging from 2 to 512 μmol/L (μM), with the final DMSO concentration maintained at ≤0.5%. Three separate control groups were included: solvent control (0.5% DMSO only), blank cell-free control, and untreated normal cell control. The cells were incubated with the compound dilutions for 48 h at 37 °C with 5% CO_2_. After incubation, the cells were washed with phosphate-buffered saline (PBS), and 110 μL of CCK-8 working solution (10% diluted in maintenance medium) was added to each well, followed by incubation for an additional 2 h. Absorbance at 450 nm (OD_450_) was measured using a microplate reader. The 20% cytotoxic concentration (CC_20_) and 50% cytotoxic concentration (CC_50_) were calculated from the absorbance data. All cytotoxicity experiments were performed in three independent biological replicates. Within each biological experiment, samples were prepared in triplicate technical wells.

$$Relative\;cell\;viability\left(\%\right)=\frac{{OD}_{450}\;of\;compound-treated\;group}{{OD}_{450}\;of\;cell\;control\;group}\times 100$$

### 2.3. In Vitro Antiviral Screening and Selectivity Index Determination

The cell seeding and culture protocols followed those outlined in Section 2.2. Cells underwent pretreatment with each compound at the specified CC_20_ concentration for 2 h before being inoculated with CV777 at a final titer of 100 TCID_50_/mL. No predefined fixed incubation time point post-infection was applied; the experiment was terminated once approximately 50% cytopathic effect (CPE) was observed in the virus-only control group. The supernatant was removed, and cell viability was assessed using the CCK-8 assay. The percentage of CPE inhibition was derived from cell viability values determined by the CCK-8 assay, rather than relying on manual microscopic scoring to quantify the extent of cytopathic lesions. This CC_20_-based screening concentration was employed to eliminate the confounding effects of compound cytotoxicity. Compounds demonstrating a viral inhibition rate greater than 50% were selected for further evaluation of the half-maximal effective concentration (EC_50_). For the measurement of EC_50_, a minimum of five serial concentration points was established for each active compound, and dose–response curves were generated using GraphPad Prism software 10.1. (GraphPad Software, San Diego, CA, USA, https://www.graphpad.com/). Dose–response curves were generated from the CPE inhibition data, and the selectivity index (SI) was calculated as CC_50_ divided by EC_50_. All antiviral screening and EC_50_-determination experiments were performed in three independent biological replicates. Within each biological experiment, samples were prepared in triplicate technical wells.

$$Virus\;inhibition\;rate\left(\%\right)=\frac{{OD}_{450}\;of\;compound-treated\;group-{OD}_{450}\;of\;virus\;control\;group}{{OD}_{450}\;of\;cell\;control\;group-{OD}_{450}\;of\;virus\;control\;group}\times 100$$

### 2.4. Viral Titer Reduction Assay (TCID_50_)

Vero E6 cells were seeded into 24-well plates at a density of 4 × 10^4^ cells per well and cultured for 12 h. The cells were then pretreated with each compound at its respective CC_20_ concentration for 2 h. A viral inoculum containing PEDV CV777 at a multiplicity of infection (MOI) of 0.1 was added directly to the medium without removing the compound. Consistent with the endpoint criterion described in Section 2.3, no predefined fixed post-infection incubation time was used. The cells were incubated continuously at 37 °C with 5% CO_2_ until approximately 50% CPE was observed in the virus-only control group. Subsequently, the cell and supernatant mixtures were collected and subjected to three freeze–thaw cycles to fully release the intracellular virions. Viral titers were determined using TCID_50_ assay, following the Reed–Muench method, on fresh Vero E6 cells seeded in 96-well plates at a density of 1 × 10^4^ cells per well. All TCID_50_ reduction experiments were performed in three independent biological replicates. Within each biological experiment, samples were set up in triplicate technical wells.

### 2.5. Indirect Immunofluorescence Assay (IFA)

Cell seeding, compound pretreatment, viral infection, and incubation were conducted as outlined in Section 2.4. Once approximately 50% CPE was observed in the virus-only control group, cells were fixed using a pre-cooled 80% acetone–PBS solution. They were then permeabilized with 0.2% Triton X-100 for 10 min at room temperature and blocked with 5% BSA at 37 °C for 1 h. Following three washes with PBST, the cells were incubated overnight at 4 °C with a mouse anti-PEDV N protein monoclonal antibody (1:2000, diluted in 5% BSA). After three additional PBST rinses, the cells were treated with FITC-conjugated goat anti-mouse IgG (1:500) for 1 h at 37 °C in the dark. Fluorescence images were then captured using a fluorescence microscope with a fixed exposure time of 15 s. IFA experiments were performed in three independent biological replicates, and representative fluorescence images are presented.

### 2.6. Dose-Dependent Antiviral Activity of Saikosaponin B2 Against PEDV

To assess the antiviral efficacy of SSB2, an integrated treatment protocol was employed, involving drug pre-incubation, viral inoculation, and sustained post-infection compound supplementation. Vero E6 cells were seeded in 24-well plates at a density of 4 × 10^4^ cells per well and cultured overnight to reach confluence. Once the cells were fully attached, they were pre-incubated for 2 h with serially diluted SSB2 in a maintenance medium composed of DMEM supplemented with 2% FBS. Following this, the cells were inoculated with PEDV CV777 at a multiplicity of infection (MOI) of 0.1. The initial medium was then replaced with fresh maintenance medium containing the same concentrations of SSB2, and the cells were cultured continuously. This process continued until approximately 50% CPE was observed in the virus-only control wells, under conditions of 37 °C and 5% CO_2_.

To quantify viral load and measure TCID_50_, cell supernatant mixtures underwent three freeze–thaw cycles to release both intracellular and extracellular virions. Total intracellular PEDV N-gene transcripts was extracted using the TIANGEN^®^ RNA extraction kit (Beijing, China). Reverse transcription quantitative PCR (qRT-PCR) targeting the PEDV N gene was then conducted, with primer sequences and reaction systems detailed in Table S1. The qRT-PCR primers targeting the PEDV N gene are designed to bind to the open reading frame of the N gene, found in both the full-length viral genomic RNA and subgenomic mRNA of PEDV. Consequently, this assay quantifies overall N-gene transcript levels without distinguishing between viral genomic RNA and subgenomic RNA. Intracellular PEDV N-gene transcript levels for all samples were calculated using a standard curve derived from serial viral RNA standards. Viral titers were determined using the TCID_50_ method as outlined in Section 2.4. All qRT-PCR experiments were performed in three independent biological replicates. Within each biological experiment, each cDNA sample was analyzed in triplicate technical replicates.

For Western blot (WB) analysis, only adherent cell monolayers were collected, and the culture supernatants were discarded. Total cellular protein was extracted using lysis buffer, omitting freeze–thaw treatment, for subsequent detection. Sample loading was normalized to β-actin grayscale values to ensure balanced protein quantities before electrophoresis. Standard procedures for SDS-PAGE, membrane transfer, blocking, and antibody incubation were followed. The primary and secondary antibodies used were consistent with those detailed in Section 2.1. IFA staining and image acquisition were conducted as described in Section 2.5, without additional cell collection. WB experiments were performed in three independent biological replicates, and representative blots are shown in the figures. Densitometric quantification was carried out on all three independent replicates.

### 2.7. Effects of Saikosaponin B2 on Different Stages of the PEDV Life Cycle

**Figure 1:**
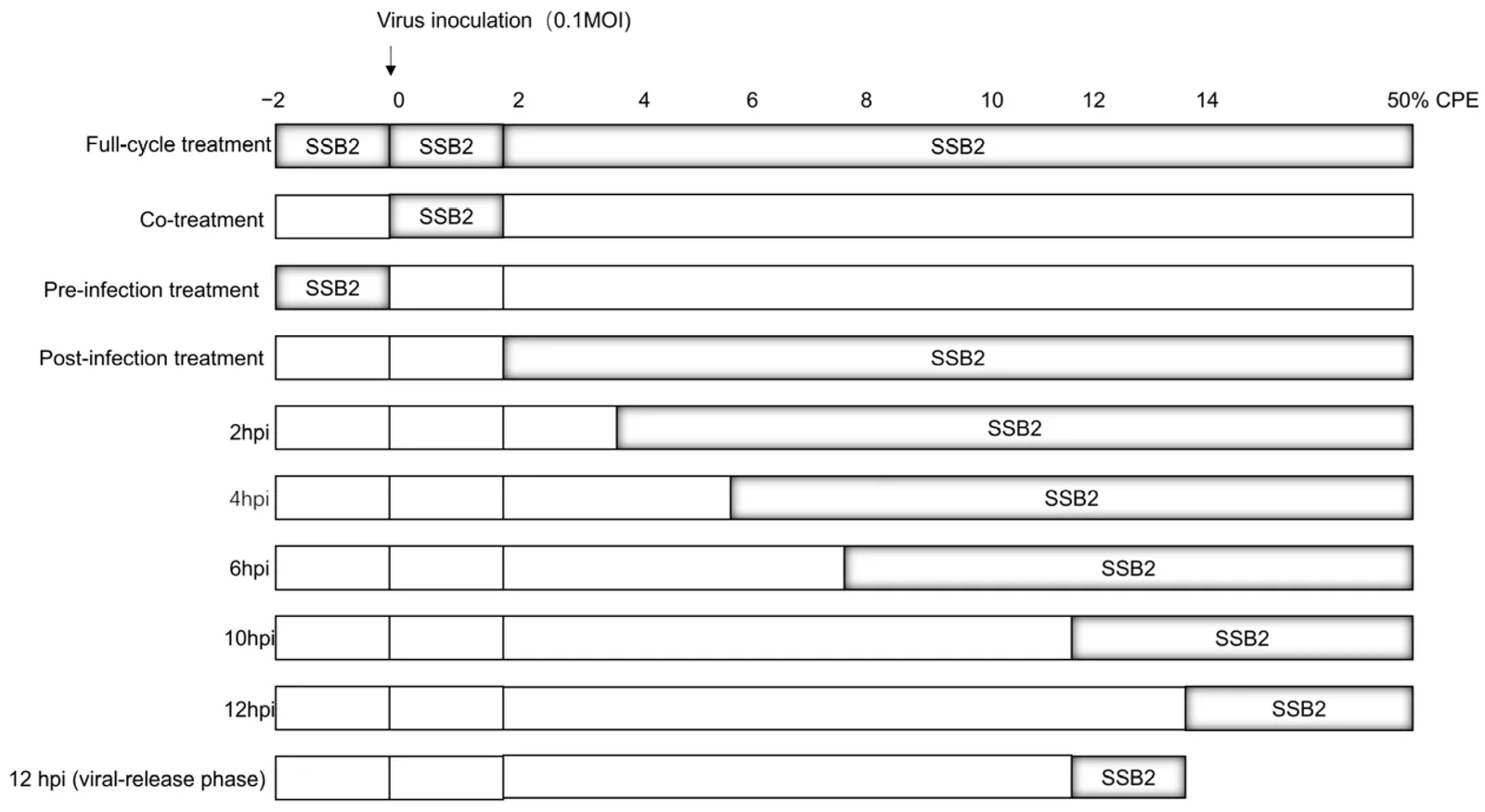
Illustrates the time-of-addition assay. The horizontal axis denotes experimental time in hours, with 0 h marking the moment of PEDV inoculation at an MOI of 0.1. Gray segments indicate incubation periods with SSB2. In the full-cycle-treatment group, SSB2 was added from −2 hpi and maintained throughout the experiment. The pre-infection-treatment group received SSB2 at −2 hpi, followed by medium replacement before viral inoculation. In the co-treatment group, SSB2 and PEDV were simultaneously added at 0 hpi. For post-infection-treatment groups, SSB2 was continuously added starting at 0, 2, 4, 6, 10, and 12 hpi, respectively. The group denoted as “12 hpi (viral-release phase)” received short-term treatment with SSB2 at 12 hpi. All cells were cultured at 37 °C with 5% CO_2_ until reaching approximately 50% cytopathic effect (CPE) compared to the virus-only-infected control wells.

A series of functional assays with standardized culture conditions were conducted to evaluate the multi-stage anti-PEDV activity of SSB2. Vero E6 cells were seeded in 24-well plates at a density of 4 × 10^4^ cells per well and cultured overnight until they reached confluence. This setup was used for various cellular drug treatment assays, including co-treatment, pre-treatment, post-infection treatment, viral adsorption, internalization, time-of-addition, and viral release assays (Figure 1). Separate virucidal tests were performed in 96-well plates. All cultures were maintained at 37 °C with 5% CO_2_ in DMEM supplemented with 2% FBS. Cell–virus co-cultures were terminated when approximately 50% CPE was observed in virus-only control wells. Mixed cell supernatant samples underwent three freeze–thaw cycles before being analyzed using parallel TCID_50_ and qRT-PCR detection methods, while only culture supernatants were collected for viral release quantification.

In the virucidal assay, PEDV CV777 (MOI = 0.1) was incubated with serially diluted SSB2 at 37 °C for 1 h. The resulting mixture was then added to pre-plated 96-well plates at a volume of 100 μL per well, with four biological replicates for each concentration. Following 2 h of viral adsorption, the unbound mixture was removed, and fresh maintenance medium was introduced. Viral inactivation efficiency was assessed using the TCID_50_ assay once the CPE endpoint was achieved.

Three cell-based intervention groups were established for the study. In the co-treatment group, cells were simultaneously incubated with PEDV and SSB2 for 2 h, then washed with PBS, and cultured until the endpoint. The pre-treatment group involved preincubating monolayers with SSB2 for 2 h, followed by a PBS rinse, PEDV inoculation for another 2 h, and continued culture until the endpoint. In the post-infection group, cells were initially infected with PEDV for 2 h, washed, and then incubated with an SSB2-containing medium. Mixed cell supernatant samples were collected for TCID_50_ and qRT-PCR analyses.

To assess early infection events, we employed adsorption and internalization assays, measuring intracellular PEDV N-gene transcript levels using qRT-PCR. In the adsorption assay, pre-chilled Vero E6 cells were co-incubated with PEDV (MOI = 0.1) and SSB2 at 4 °C for 2 h. Following this, unbound virions were removed by washing with pre-cooled PBS, and the cell pellets were collected for quantifying the PEDV N gene. For the internalization assay, cells were first incubated with PEDV at 4 °C for 2 h to facilitate binding. After the free virus was removed, pre-chilled SSB2 medium was added, and the plates were incubated at 37 °C for 2 h to enable viral penetration. Surface-attached virions were neutralized with acidic PBS (pH 3.0), and the cells were thoroughly washed and harvested for detecting intracellular viral RNA.

In the time-of-addition assay, SSB2 was added at intervals of 2, 4, 6, 10, and 12 h following viral adsorption. The cultures were maintained until reaching the CPE endpoint, after which cell–supernatant mixtures were collected for combined qRT-PCR and TCID_50_ analysis. Cells were infected with PEDV for viral release detection. Following a 2 h virus adsorption period, the inoculum was eliminated, and cells were cultured for an additional 10 h to facilitate intracellular viral replication and virion formation. Afterward, the supernatant was removed, and cell monolayers were rinsed twice with sterile PBS. Subsequently, fresh maintenance medium supplemented with SSB2 was introduced, and cells were cultured for 2 h. Finally, culture supernatants were collected for extracellular virion quantification through TCID_50_ and qRT-PCR analyses.

### 2.8. Validation of the Broad-Spectrum Antiviral Activity Against PEDV Field Epidemic Strains

To assess the broad-spectrum anti-PEDV efficacy of SSB2, we tested two prevalent field strains, JL-06/2023 (G2a subtype) and JL-08/2023 (G2b subtype). Vero E6 cells were seeded in 24-well plates at a density of 4 × 10^4^ cells per well and cultured overnight until they reached full confluence. The cells were pretreated with serial dilutions of SSB2 according to the pretreatment and continuous administration protocol outlined in Section 2.6. Subsequently, they were infected with each clinical isolate at an MOI of 0.1. Throughout the incubation, the medium was supplemented with the compound. Cultures were maintained at 37 °C with 5% CO_2_ until approximately 50% CPE was observed in the virus-only control wells. At the experimental endpoint, the cell–supernatant mixtures were collected and subjected to three freeze–thaw cycles. Viral titers were measured using the TCID_50_, qRT-PCR, IFA, and WB method. The inhibitory effectiveness of SSB2 against the two clinical strains was then compared to its activity against the prototype CV777 strain.

### 2.9. Statistical Analysis

All experiments included at least three independent biological replicates, with data presented as the mean ± standard deviation (SD). For multi-group statistical analyses, we employed one-way analysis of variance (ANOVA) followed by Tukey’s multiple-comparison test using GraphPad Prism. Statistical significance thresholds were established as follows: * *p* < 0.05, ** *p* < 0.01, *** *p* < 0.001, **** *p* < 0.0001; *p* ≥ 0.05 indicated no statistically significant difference (ns).

## 3. Results

### 3.1. In Vitro Antiviral Screening and Selectivity Index of the Candidate Compound

**Figure 2:**
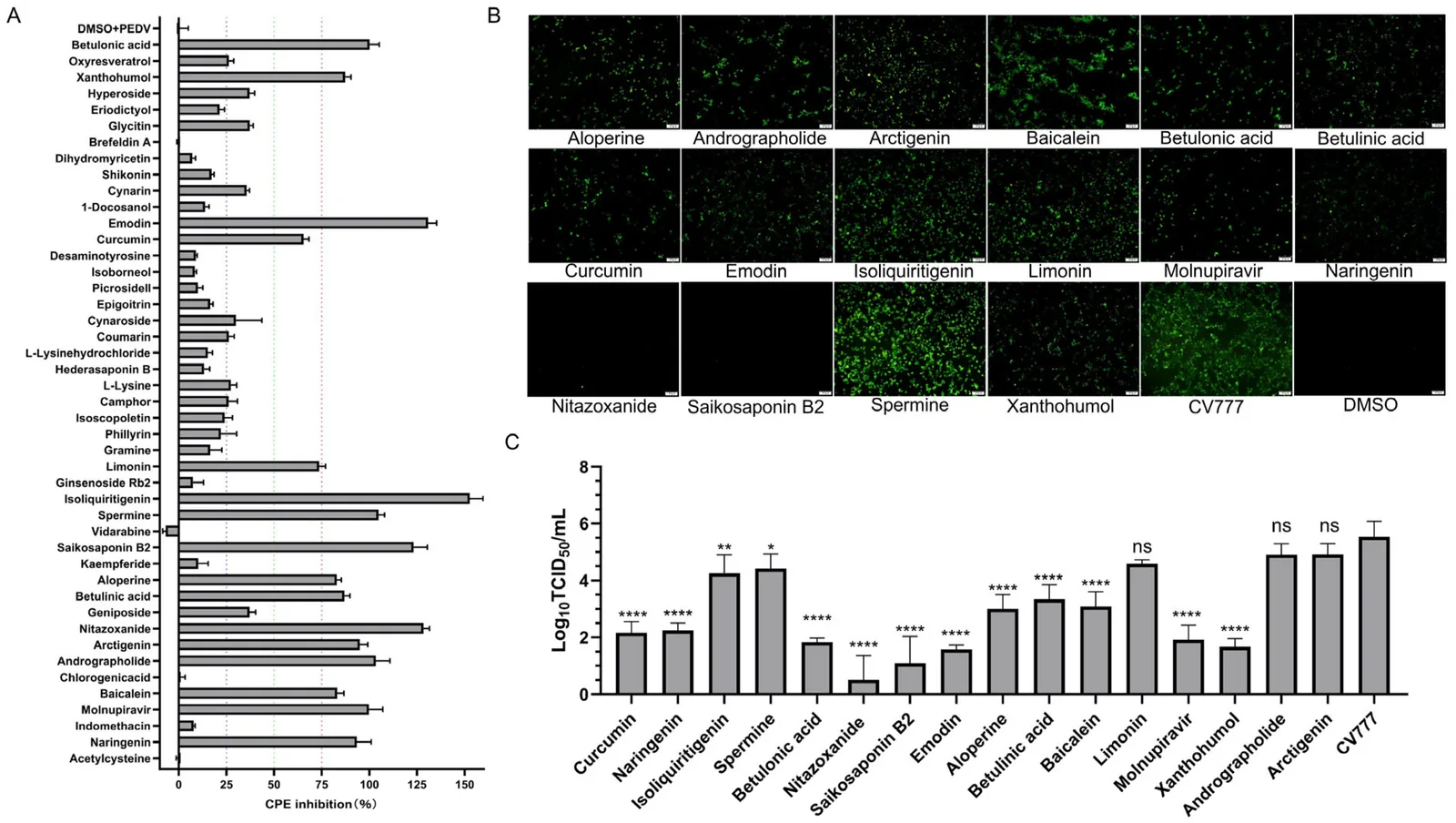
Screening and functional validation of small-molecule compounds against PEDV. (**A**) Initial screening of 45 candidate small-molecule drugs was conducted based on the CPE. CPE-inhibition percentages were calculated from CCK-8-determined cell-viability values. The length of the horizontal bars indicates the percentage inhibition of PEDV-induced CPE, and each compound was pre-treated at its respective CC_20_ concentration for 2 h before virus inoculation and kept in the culture medium throughout the whole incubation period. This unified treatment regimen was applied to all panels (**A**–**C**) of Figure 1. Compounds showing over 50% inhibition identified as primary positive hits. (**B**) IFA was used to verify the activity of selected compounds. Green fluorescence indicates intracellular expression of the PEDV nucleoprotein. DMSO-treated cells served as the infected solvent control, uninfected Vero E6 cells were the blank negative control, and CV777-infected cells without compound treatment acted as the virus-positive control. All microscopic fields have scale bars set at 100 μm. (**C**) The TCID_50_ assay quantitatively detected infectious progeny virions. The vertical axis shows log_10_ TCID_50_/mL, where lower values indicate stronger inhibition of viral proliferation. Asterisk (*) indicates a significant difference among them. (* *p* < 0.05, ** *p* < 0.01, **** *p* < 0.0001). “ns” is used to indicate lack of statistical significance.

We initially confirmed that 0.5% DMSO was non-toxic to Vero E6 cells. The cytotoxicity profiles, CC_20_ and CC_50_ for all 45 candidate compounds were assessed (Supplementary Table S2). In CPE inhibition screening, 16 compounds, including curcumin, naringenin, molnupiravir, baicalein, isoliquiritigenin, andrographolide, arctigenin, nitazoxanide, betulinic acid, aloperine, emodin, limonin, spermine, SSB2, xanthohumol, and betulonic acid, demonstrated over 50% suppression of PEDV-induced lesions (Figure 2A). Nine other compounds, such as geniposide and hyperoside, exhibited mild antiviral activity with less than 50% CPE inhibition. The EC_50_ and SI for the 16 effective compounds were calculated (Table 1 and Supplementary Figure S1). Andrographolide, nitazoxanide, SSB2, xanthohumol, and betulonic acid showed high SI values (>5), indicating broad therapeutic windows. Curcumin, naringenin, isoliquiritigenin, betulinic acid, aloperine, and emodin had SI values greater than 1. Molnupiravir and baicalein exhibited minimal cellular toxicity, with low CC_50_ values limiting precise SI calculation, though their selectivity was acceptable. IFA targeting the PEDV N protein showed intense green fluorescence in the virus-only CV777 control group (Figure 2B), while most compound-treated groups had significantly reduced viral antigen signals. Spermine and isoliquiritigenin only weakly suppressed viral protein expression. Subsequent viral titration using the TCID_50_ assay quantified progeny virus loads (ordinate: Log_10_ TCID_50_/mL, Figure 2C). Compounds like curcumin, naringenin, molnupiravir, nitazoxanide, emodin, SSB2, betulinic acid, aloperine, baicalein, and betulonic acid significantly reduced PEDV titers. However, limonin, andrographolide, and arctigenin showed non-significant antiviral effects.

### 3.2. Dose-Dependent Anti-PEDV Activity of the Selected Compound

**Figure 3:**
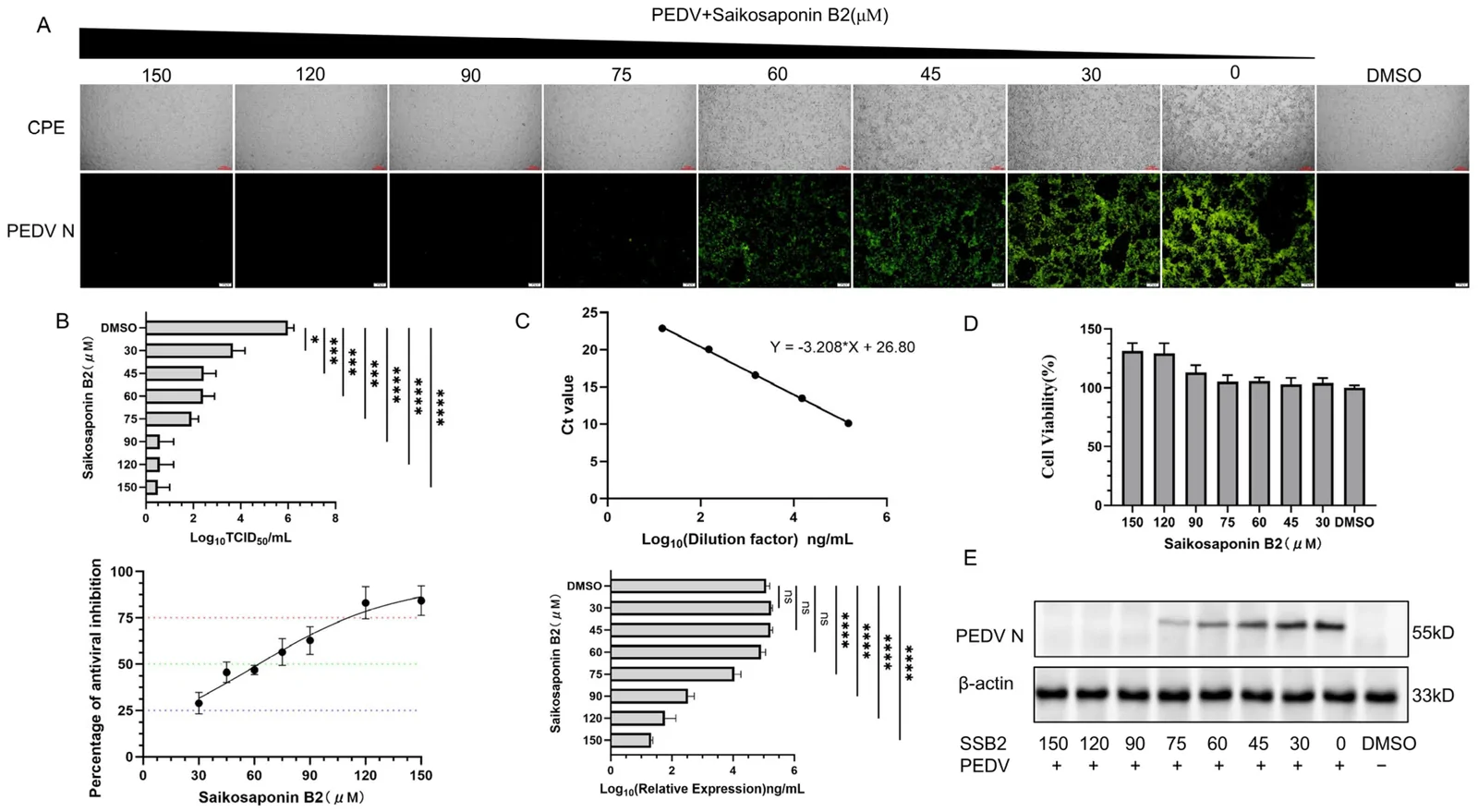
Illustrates the dose-dependent antiviral efficacy of SSB2 against PEDV. In all experiments depicted in Figure 2, Vero E6 cells underwent a 2 h pretreatment with SSB2 before PEDV infection. SSB2 was consistently present in the culture medium during the entire incubation period. No specific post-infection time point was designated; sample collection occurred upon reaching approximately 50% cytopathic effect compared to the virus-only control group. (**A**) The panel shows bright-field CPE micrographs and IFA images targeting the PEDV N protein, indicated by green fluorescence. Vero E6 cells were treated with serial dilutions of SSB2, ranging from 0 to 150 μM, while DMSO served as the virus-infected solvent control. Scale bars are set at 200 μm for bright-field CPE images and 100 μm for all IFA micrographs. (**B**) This section includes TCID_50_ quantification and a dose–response inhibition curve. The upper bar chart displays log_10_TCID_50_/mL viral titers under varying SSB2 concentrations. The lower curve illustrates the percentage of antiviral inhibition relative to SSB2 concentration. Statistical significance is denoted as follows: ns for non-significant results. (**C**) qRT-PCR was used to detect PEDV N gene transcripts. The upper panel features a standard curve for absolute viral nucleic acid quantification, while the lower bar plot shows log_10_-transformed relative intracellular viral RNA levels. (**D**) Cytotoxicity of SSB2 against Vero E6 cells. Cell viability was determined by the CCK-8 assay after 48 h treatment with SSB2 (30–150 μM). DMSO served as solvent control. (**E**) Western blot analysis of PEDV N-protein expression. Immunoblot bands of PEDV-N and β-actin (loading control) under SSB2 treatment (30–150 μM). Asterisk (*) indicates a significant difference among them. (* *p* < 0.05, *** *p* < 0.001, **** *p* < 0.0001). “ns” is used to indicate lack of statistical significance.

We initially assessed the dose-dependent anti-PEDV activity of SSB2 in Vero E6 cells. Cells infected with PEDV were treated with serial dilutions of SSB2, ranging from 0 to 150 μM. Observations of CPE and IFA targeting the PEDV nucleoprotein showed that SSB2 alleviated PEDV-induced cytopathy and reduced viral N protein fluorescence in a concentration-dependent manner. Significant viral suppression was evident at 75 μM, with only minimal residual fluorescence, while near-complete elimination of viral antigen signals occurred at 120 and 150 μM (Figure 3A). The TCID_50_ assay confirmed the dose-responsive antiviral efficacy of SSB2 (Figure 3B). The upper bar graph illustrates the log_10_-transformed titers of infectious progeny virions, which decreased significantly with increasing SSB2 concentrations. The lower dose–response curve of the antiviral inhibition rate was used to determine the EC_50_ at 59.80 μM, highlighting a positive correlation between SSB2 dosage and inhibitory efficiency. A notable discrepancy in EC_50_ was observed when comparing this value with our preliminary screening data. The EC_50_ of 11.55 μM, as presented in Table 1, was derived from CCK-8-measured cell viability in PEDV-infected cells, while the corresponding SI of 19.77 was calculated from CCK-8-derived CC_50_ data. This difference can be attributed to the distinct experimental endpoints: CCK-8 assesses cell-protective effects, whereas TCID_50_ quantifies the production of infectious progeny virus. Consequently, subsequent mechanistic experiments utilized the TCID_50_-derived EC_50_. Quantification of intracellular PEDV N-gene transcripts loads was performed using qRT-PCR (Figure 3C). A standard curve for absolute viral nucleic acid quantification was constructed with the regression equation Y = −3.208X + 26.80 and a high correlation coefficient of R^2^ = 0.990. The bar graph of relative PEDV N gene expression indicates that SSB2 progressively reduced viral RNA abundance as the concentration increased. No statistically significant reduction in viral N-gene transcripts was observed at 30, 45, and 60 μM. However, starting from 75 μM and up to 150 μM, SSB2 significantly down regulated viral RNA levels compared to the virus-only control group, with continuous declines in viral transcript abundance across the 75–150 μM concentration range. To assess the cytotoxic impact of SSB2 on Vero E6 cells, cell viability was assessed using the CCK-8 assay following exposure to varying concentrations of SSB2 (30, 45, 60, 75, 90, 120, and 150 μM). Figure 3D illustrates that there was no significant decrease in cell viability at any of the tested doses. Cell viability remained above 100% even at the highest concentration of 150 μM, suggesting that SSB2 demonstrated minimal cytotoxicity towards Vero E6 cells within this concentration range. The solvent control utilized was DMSO. WB analysis supported the transcriptional findings at the protein level (Figure 3E). PEDV N protein (55 kD) expression was normalized to β-actin (33 kD) as the internal reference. Gradual SSB2 treatment reduced viral N protein accumulation, with the most substantial inhibitory effect observed at 150 μM SSB2. Overall, multiple phenotypic and molecular assays confirmed that SSB2 exhibits strong concentration-dependent anti-PEDV activity.

### 3.3. SSB2 Inhibits Early Steps of the PEDV Infection Cycle, Including Viral Adsorption and Internalization

**Figure 4:**
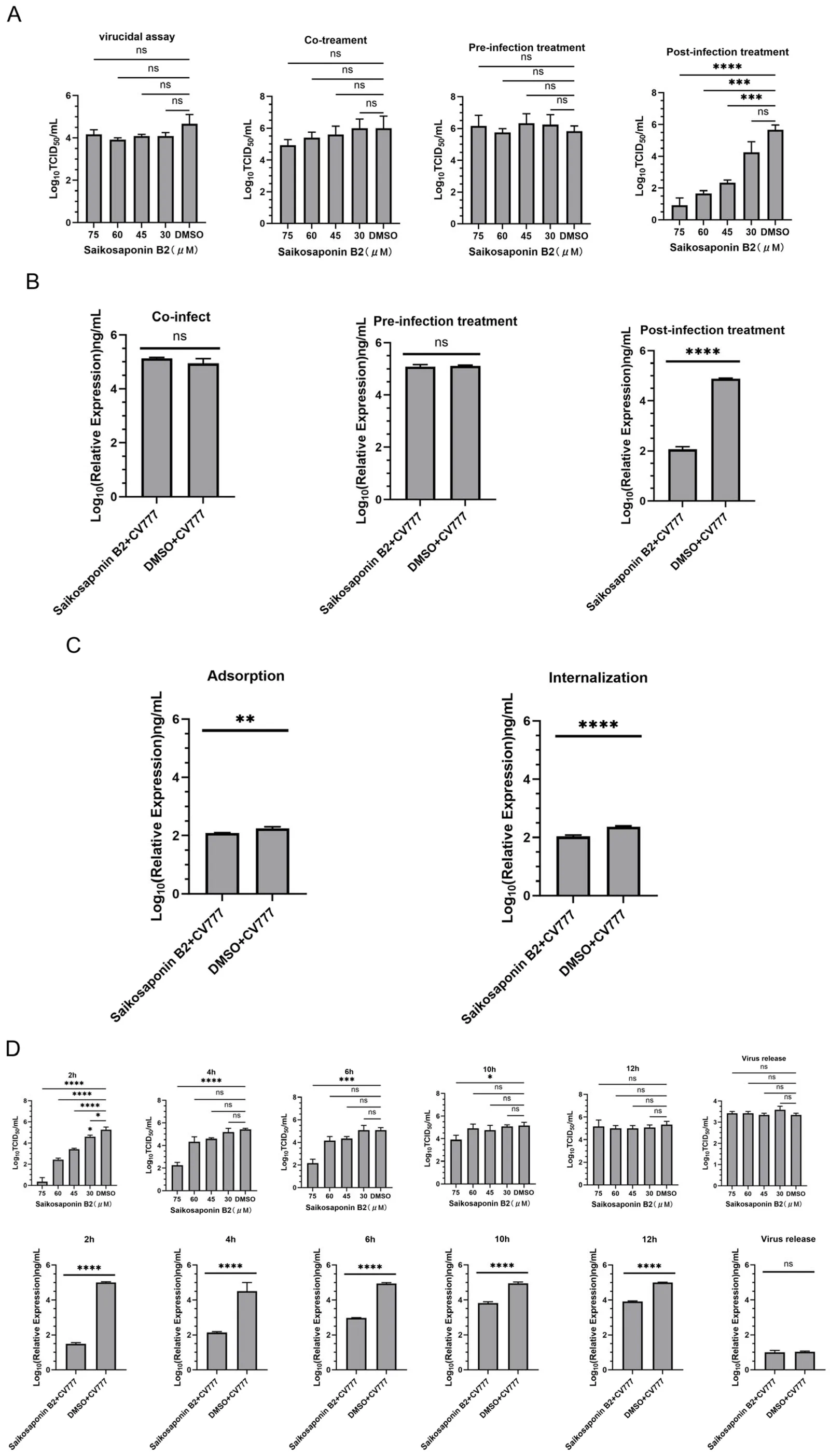
Mechanistic investigation of SSB2 against PEDV using multi-stage time-of-addition and viral entry assays. (**A**) TCID_50_ viral titer measurements are provided for four treatment regimens: virucidal assay, co-treatment, pre-infection treatment, and post-infection treatment, all with serial dilutions of SSB2 (30–75 μM). The vertical axis displays log_10_TCID_50_/mL. (**B**) Relative PEDV N-gene transcripts expression is assessed via qRT-PCR under co-infection, pre-infection, and post-infection SSB2 treatments at a fixed concentration of 75 μM. Data are shown as log_10_-transformed relative viral transcript abundance. Panel (**A**) measures infectious virus titers, whereas panel (**B**) validates antiviral activity at the viral RNA level. (**C**) qRT-PCR quantification of intracellular viral RNA evaluates SSB2’s effects on PEDV adsorption and internalization during viral entry, using 75 μM SSB2. (**D**) The time-course kinetic analysis spans from 2 to 12 hpi. Upper panels depict log_10_TCID_50_/mL infectious viral titers at sequential time points; lower panels show relative PEDV N gene mRNA levels quantified by qRT-PCR. The far-right subgraph presents independent viral release assay results. Asterisk (*) indicates a significant difference among them. (* *p* < 0.05, ** *p* < 0.01, *** *p* < 0.001, **** *p* < 0.0001). “ns” is used to indicate lack of statistical significance.

To further investigate the antiviral mechanism of SSB2 against PEDV, we conducted time-of-addition assays to identify the specific stage in the viral life cycle that SSB2 targets. TCID_50_ quantification indicated no statistically significant reduction in PEDV infectivity for the virucidal, co-infection, and pre-infection treatment groups. However, post-infection treatment with SSB2 resulted in a dose-dependent suppression of infectious viral progeny. Significant inhibition was observed at concentrations of 45, 60, and 75 μM, whereas 30 μM SSB2 did not exhibit noticeable antiviral activity (Figure 4A).

In alignment with the viral titer results, qRT-PCR analysis of intracellular viral N-gene transcripts revealed that SSB2 did not inhibit PEDV during co-infection or pre-infection incubation. However, it significantly reduced viral RNA accumulation following viral entry in the post-infection stage (Figure 4B).

Additional assays focused on the early entry cascade confirmed that SSB2 significantly impeded both viral adsorption and subsequent internalization (Figure 4C).

Time-course kinetic experiments spanning 2 to 12 h post-infection (hpi) elucidated the antiviral dynamics of SSB2 (Figure 4D). TCID_50_ titration of released infectious virions demonstrated significant suppression at the very early phase (2 hpi). This dose-dependent inhibition remained significant at 4 and 6 hpi, but by 10 and 12 hpi, all treatment groups showed no statistical inhibitory effects. In contrast, qRT-PCR quantification of intracellular PEDV N transcripts indicated a sustained and strong suppression of N-gene transcript accumulation from 2 to 12 hpi. The dedicated release assay showed no significant blockade of viral release.

These multi-stage functional assays collectively demonstrated that SSB2 primarily combats PEDV infection by blocking viral adsorption and internalization during the entry phase and suppressing intracellular N-gene transcript accumulation in the early post-infection phase. SSB2 does not exhibit neutralizing activity against extracellular virions and does not interfere with late-stage viral release.

### 3.4. Broad-Spectrum Activity Against Clinical PEDV Epidemic Strains

**Figure 5:**
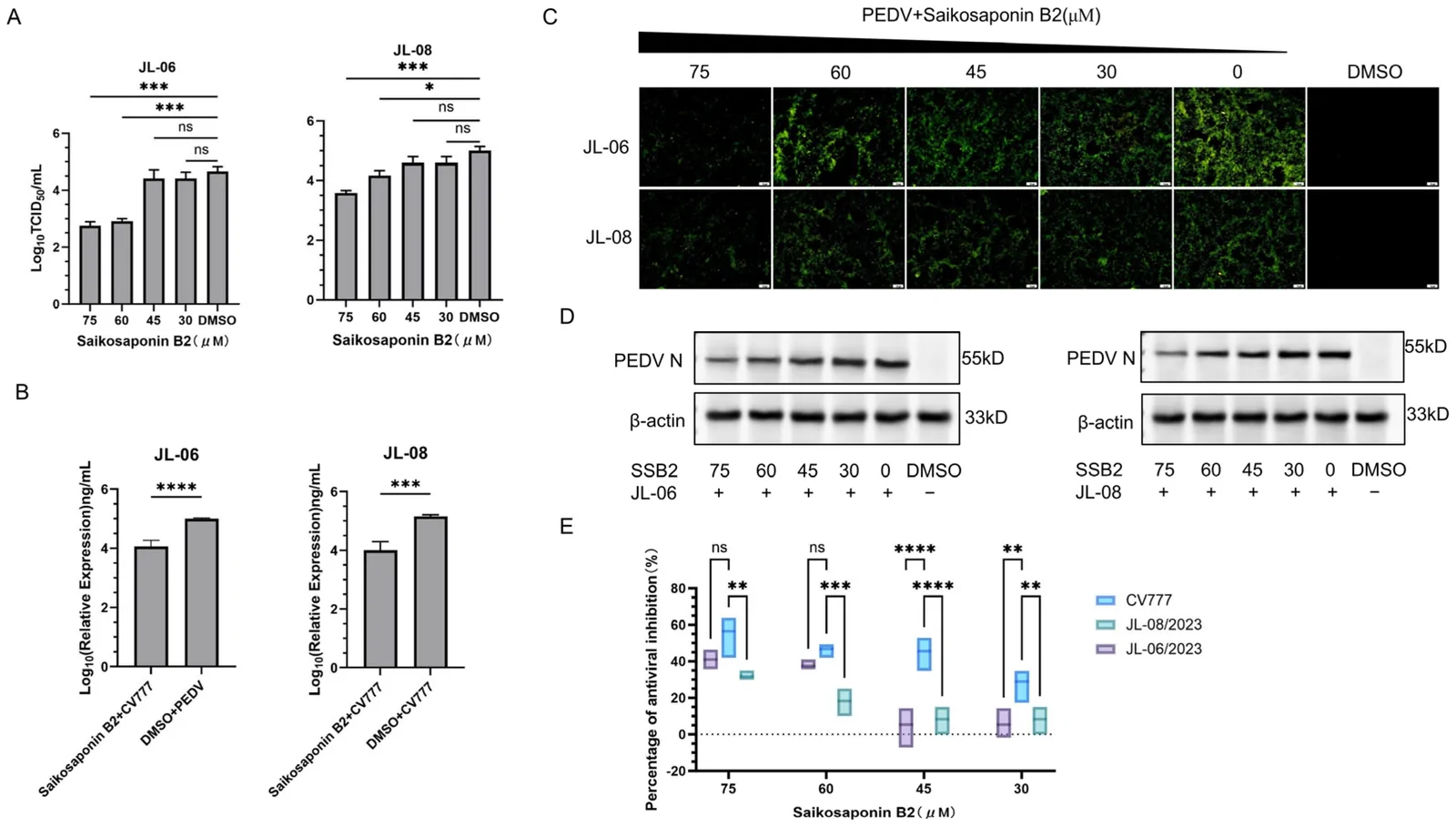
Illustrates the broad-spectrum antiviral efficacy of SSB2 against two prevalent G2-subtype PEDV clinical isolates. (**A**) The TCID_50_ quantification of infectious progeny virions is shown. Vero E6 cells, infected with either JL-06 (G2a) or JL-08 (G2b) at an identical MOI of 0.1, were treated with varying concentrations of SSB2 (ranging from 30 to 75 μM), while DMSO-treated infected cells served as the viral control. The vertical axis indicates log_10_TCID_50_/mL. (**B**) RT-qPCR analysis of viral RNA levels of two field-isolated PEDV strains (JL-06 and JL-08) after treatment with 75 μM SSB2. (**C**) Immunofluorescence assay (IFA) detection of PEDV N-protein. Viral-specific fluorescence signals were observed under fluorescence microscopy. Scale bar = 100 μm. (**D**) Western blot analysis of PEDV-N protein expression. The displays immunoblot bands of the viral N protein and β-actin (loading control) following treatment with SSB2 at concentrations of 30, 45, 60, and 75 μM. (**E**) Box plots display the percentage of antiviral inhibition by SSB2 across the classical CV777 (G1), JL-06 (G2a), and JL-08 (G2b) strains, all inoculated at MOI = 0.1, at serial concentrations of 30, 45, 60, and 75 μM. Asterisk (*) indicates a significant difference among them. (* *p* < 0.05, ** *p* < 0.01, *** *p* < 0.001, **** *p* < 0.0001). ns” is used to indicate lack of statistical significance.

Serially diluted SSB2 was administered to Vero E6 cells infected with two prevalent G2-subtype PEDV clinical isolates, JL-06 (G2a) and JL-08 (G2b), to evaluate antiviral activity through multiple detection methods (Figure 4A–D). Infectious progeny viral titers were quantified initially using the TCID_50_ assay, with DMSO-treated infected cells serving as the virus-only control (Figure 5A). The log_10_-transformed TCID_50_/mL titers for both strains exhibited a progressive decrease as SSB2 concentrations increased. Significant reductions in infectious viral titers were observed at 60 and 75 μM, while no notable inhibitory effects were detected at 30 and 45 μM for either field isolate. In line with the reduction in infectious virus yields, RT-qPCR analysis indicated a marked decrease in intracellular viral RNA levels following treatment with 75 μM SSB2 (Figure 5B). Immunofluorescence staining further demonstrated a concentration-dependent attenuation of PEDV N-protein fluorescence signals across the 30–75 μM SSB2 gradient (Figure 5C). Consistent with these findings, Western blot assays revealed a gradual decline in the abundance of intracellular viral N-protein bands as SSB2 concentrations increased for both JL-06 and JL-08 (Figure 5D).

Figure 5E displays box plots comparing the percentage of antiviral inhibition by SSB2 against the classical prototype CV777 (G1), JL-06 (G2a), and JL-08 (G2b) strains. At concentrations of 75 and 60 μM, pairwise comparisons revealed statistically significant differences in inhibitory efficiency among the three strains. When the concentration was reduced to 45 μM, the antiviral activity of SSB2 against JL-06 and JL-08 decreased sharply, showing extremely significant differences compared to CV777. However, no significant difference was noted between JL-06 and JL-08 at this concentration. At 30 μM, the inhibition ratios for JL-06 and JL-08 nearly reached zero, yet significant differences in antiviral potency persisted between CV777 and the two clinical isolates.

These data collectively demonstrate that SSB2 exhibits dose-dependent anti-PEDV activity against both G2a and G2b field isolates. However, its inhibitory efficacy against variant strains is significantly weaker compared to its effectiveness against the classical CV777 prototype strain at equivalent concentrations.

## 4. Discussion

In recent years, variant PEDV strains have persistently circulated, diminishing the protective efficacy of commercial live-attenuated vaccines and resulting in high piglet mortality and significant economic losses in the pig industry. The development of small-molecule antiviral agents has emerged as a promising strategy for PEDV prevention and control [22]. In this study, we conducted an in vitro anti-PEDV screening of 45 small-molecule compounds. The primary screening identified 16 compounds with notable inhibitory effects on virus-induced cytopathic lesions. Among these, SSB2 exhibited a SI of 19.77, significantly higher than most hits, including curcumin and naringenin, indicating favorable biosafety and a broad therapeutic window [23,24]. Although nitazoxanide also demonstrated strong antiviral potency, SSB2, the primary bioactive component of the traditional Chinese medicinal herb Bupleuri Radix, offers advantages in raw material accessibility and translational research potential. Minor discrepancies in the antiviral performance of hyperoside and coumarin compared to previous studies may be due to differences in viral strains and compound extraction protocols across independent research [25,26].

Divergent results occasionally appeared in our functional assays involving qRT-PCR, Western blot, and TCID_50_ measurements. The qRT-PCR assay quantifies intracellular viral RNA transcripts, detectable even when downstream viral-protein translation or virion assembly is compromised. Western blot analysis indicates the abundance of translated viral structural proteins, while TCID_50_ represents a stringent endpoint detecting only mature, infectious progeny virions. These inherent differences in measured endpoints may elucidate the partial mismatch among the datasets, suggesting that SSB2 may induce post-transcriptional inhibitory effects, diminishing viral-protein production and infectious-virus yields without completely degrading intracellular viral RNA [27,28]. Combined nucleic acid and protein quantification consistently showed that SSB2 reduces intracellular viral RNA levels, blocks viral N protein accumulation, decreases infectious progeny virion yields, and mitigates PEDV-induced cellular lesions. Given the conserved replication machinery among coronaviruses, we inferred that SSB2 likely interferes with viral transcription and translation. In line with previous reports that pentacyclic triterpenoids have broad anti-coronavirus effects [15], our data expand the functional evidence of triterpenoid saponins against coronaviruses and support further mechanistic research targeting viral transcriptional and translational regulation.

Virus inactivation tests, multi-regimen drug administration, and time-of-addition assays were conducted to identify the specific functional stage of SSB2 within the PEDV life cycle. Virucidal assays confirmed that SSB2 does not directly inactivate extracellular virions, as evidenced by the unchanged viral RNA abundance and progeny titers in co-treatment groups. This distinguishes SSB2 from flavonoid derivatives, which rapidly disrupt coronavirus lipid envelopes upon co-incubation [29], and from synthetic small-molecule antivirals that primarily target intracellular viral replicative enzymes [30]. Compounds targeting different viral cycle stages may produce synergistic antiviral effects when co-administered, warranting further exploration in future studies [31]. Single pre-infection dosing showed no protective effects against PEDV, indicating that SSB2 cannot stably bind to cell surface receptors or establish a persistent intracellular antiviral state. Attachment and internalization assays confirmed that SSB2 blocks viral entry, but this inhibitory activity depends on continuous exposure to the compound and is completely lost after drug washout. This reversible suppressive property has been documented in prior research on SSB2 [18,19]. Glycyrrhetinic acid, another pentacyclic triterpenoid analog, exhibits a similar mode of action [32], explaining the lack of antiviral activity in the pre-treatment single-dosing group. Comparisons of different treatment regimens revealed that post-infection intervention induced stronger antiviral effects than continuous full-cycle drug incubation. We speculate that prolonged SSB2 exposure may disrupt host cell proliferative homeostasis, partially offsetting its antiviral efficacy, as chronic treatment with natural small molecules has been shown to interfere with normal cellular physiological balance [33]. Short-term post-infection dosing causes less disruption to cell homeostasis and retains the antiviral potency of SSB2. Time-of-addition kinetic experiments further demonstrated that the antiviral capacity of SSB2 declines with delayed drug supplementation, showing strict time dependence and concentrating its core inhibitory window in the early phase of infection. Notably, TCID_50_ quantification of released infectious virions showed significant suppression only at 2–6 h post-infection, while intracellular viral RNA was consistently downregulated from 2 to 12 hpi. Viral release assays confirmed that SSB2 does not significantly inhibit progeny virion egress. Collectively, SSB2 hinders PEDV entry and intracellular viral RNA accumulation during early and mid-stage infection during early and mid-stage infection, consistent with the nucleic acid and protein phenotypic data described above. Subsequent cell proliferation functional assays are needed to validate this conjecture and clarify the molecular mechanisms responsible for differential antiviral efficacy across distinct administration schedules.

Accumulating evidence suggests that SSB2 exhibits broad-spectrum antiviral activity against various RNA viruses and coronaviruses. In addition to its known functions in regulating host inflammation and suppressing viral RNA accumulation, our research has confirmed that SSB2 effectively impedes PEDV entry, particularly during the internalization phase [34]. This inhibitory mechanism during entry aligns with prior studies on feline herpesvirus, illustrating SSB2’s interference with spike-protein-mediated viral invasion [18]. Comparable conserved antiviral properties have also been noted in SARS-CoV-2 and hepatitis C virus [19,20,21,35]. While SSB2 may display preferential targeting depending on the virus, these collective findings establish SSB2 as a natural viral entry inhibitor with broad-spectrum activity. Consequently, this monomer derived from Chinese medicine emerges as a promising candidate for the development of broad-spectrum antiviral agents.

To evaluate the broad-spectrum antiviral potency of SSB2, we tested its effects on two prevalent field variant strains, JL-06 (G2a) and JL-08 (G2b), in addition to the classical prototype CV777 strain. Continuous amino acid mutations in the spike protein of circulating domestic PEDV strains often reduce the effectiveness of commercial vaccines and small-molecule antiviral candidates [36]. Our findings demonstrated that SSB2 exhibited dose-dependent inhibitory activity against both G2 subtype isolates, with viral genomic mutations unable to completely evade SSB2’s suppressive effects. This provides practical in vitro evidence for controlling variant PEDV epidemics on pig farms. A comparative analysis showed that SSB2’s inhibitory activity was slightly weaker against JL-08 than JL-06. This difference likely stems from distinct amino acid mutations in key functional domains of the spike protein, which may affect SSB2’s efficiency in blocking viral attachment [37]. However, mutations related to viral membrane fusion or intracellular replication might also contribute to this strain-specific difference, as SSB2 targets both viral entry and post-entry replication steps. Strain-specific differences can be ascribed to intrinsic variations in viral entry efficiency, replication kinetics, and virulence across different PEDV isolates. The modified replication traits and host factor reliance of JL-08 might diminish the post-entry inhibitory effectiveness of SSB2. Additional comparative research is necessary to elucidate the molecular mechanism behind this varying antiviral impact. Box plot data indicate that SSB2’s antiviral inhibition rate against CV777 was significantly higher than against JL-06 and JL-08 at the same concentrations. While most reported pentacyclic triterpenoids maintain stable antiviral activity against a single coronavirus strain, SSB2 consistently inhibits multiple circulating PEDV variants, showing promise in addressing viral genetic variation. It is important to note that all broad-spectrum assessments were conducted in vitro, without supporting in vivo animal efficacy data. Nonetheless, these results establish SSB2 as a broad-spectrum lead compound for anti-PEDV research and provide a solid cellular experimental basis for developing novel antivirals targeting mutated PEDV strains.

The effective antiviral concentrations of SSB2 in the current Vero-E6-based assay ranged from 75 to 150 μM for CV777 and 60 to 75 μM for field isolates JL-06 and JL-08. Caution is warranted when interpreting these in vitro concentrations in the context of pharmacological feasibility, as they may not accurately represent actual drug exposure in porcine intestinal tissue in vivo. A theoretical uniform-distribution calculation, based on a previously reported oral SSB2 dose of 25 mg·kg^−1^ in mice, estimated a maximum systemic concentration of approximately 51.45 μM; however, this figure serves only as an upper-limit estimation rather than a measured plasma concentration [38]. Due to the low oral bioavailability of saikosaponins, actual plasma levels are anticipated to be significantly lower. Nonetheless, local accumulation within the gastrointestinal lumen could potentially achieve the 60 to 75 μM threshold at the intestinal mucosal surface, the primary site of PEDV infection. Physiological differences between mice and piglets hinder direct inter-species extrapolation, necessitating further porcine pharmacokinetic studies to determine whether SSB2 can attain therapeutically relevant concentrations at infection sites.

Integrating all in vitro cellular experimental data, SSB2 exhibits a distinctive pentacyclic triterpenoid structure that allows it to simultaneously interfere with PEDV entry and intracellular viral replication, maintaining stable inhibitory potency against circulating field variants JL-06 and JL-08. However, this study has two significant limitations that warrant discussion. First, all antiviral functional assays were conducted on Vero E6 cells, which do not fully replicate the intestinal physiological microenvironment during native PEDV infection as effectively as porcine intestinal epithelial cell lines like IPEC-J2. Future research will verify the anti-PEDV efficacy of SSB2 on IPEC-J2 cells, and in vivo piglet challenge trials will assess its actual protective efficacy at the animal level. Second, while this research systematically characterized the antiviral phenotypic properties of SSB2, its direct molecular binding targets remain unidentified. Consequently, the molecular basis for variable efficacy across different drug administration schedules and the potential interaction between SSB2 and host cell proliferation pathways remain unclear. Subsequent research will employ targeted experiments such as molecular docking, co-immunoprecipitation (Co-IP), surface plasmon resonance (SPR), and multi-omics profiling to elucidate the precise molecular regulatory pathways of SSB2. Additionally, two overlooked areas merit further exploration: the long-term cytotoxicity and metabolic stability of SSB2 were not assessed in the current study, and the synergistic antiviral potential of SSB2 in combination with other anti-PEDV agents was not explored, both of which can be addressed in future studies.

## 5. Conclusions

In summary, SSB2 is a pentacyclic triterpenoid saponin with dual anti-PEDV effects, blocking both viral adsorption and internalization while also inhibiting intracellular viral replication. It maintains strong inhibitory activity against the prevalent field strains JL-06 and JL-08. Therefore, SSB2 is a promising candidate for developing anti-PEDV therapeutics. However, several significant limitations related to this in vitro investigation must be considered, as detailed in Section 4.

## Figures and Tables

**Table 1:** Antiviral activity EC_50_ and SI of the 16 potent hits.

Name	EC_50_ (μM)	SI (CC_50_/EC_50_)
Curcumin	59.90	1.22
Naringenin	146.8	1.88
Molnupiravir	29.63	/
Baicalein	174.60	/
Isoliquiritigenin	21.93	2.81
Andrographolide	13.21	12.68
Arctigenin	268.70	/
Nitazoxanide	2.56	145.23
Betulinic acid	176.00	1.40
Aloperine	164.80	1.86
Emodin	31.49	2.66
Limonin	278.80	/
Spermine	69.40	/
Saikosaponin B2	11.55	19.77
Xanthohumol	3.10	5.24
Betulonic acid	8.70	7.82

## Data Availability

The original contributions presented in this study are included in the article. Further inquiries can be directed to the corresponding authors.

## Supplementary Materials

The following supporting information can be downloaded at: https://www.mdpi.com/article/10.3390/v18090997/s1, Table S1: Porcine epidemic diarrhea virus real-time RT-PCR primers and probe sequences and system configuration; Table S2: Cytotoxicity to Vero E6 cells; Figure S1: Determination of EC_50_ values of candidate compounds against PEDV infection.
