# Supplementary material for: In Vitro Assessment of Antiviral Activity of Saikosaponin B2 Against Porcine Epidemic Diarrheal Virus
Source: Viruses. 2026 Sep 10;18(9):997. doi: 10.3390/v18090997 (PMC13612102; doi:10.3390/v18090997)
Supplement: Supplementary file 1 [file viruses-18-00997-s001.zip › Supplementary Table S1.pdf]

Table S1.1 Porcine epidemic diarrhea virus real-time RT-PCR primers and probe sequences

Target gene	Primer name	Sequence (5′–3′)(AF353511.1)	Position	Tm (℃)	Amplicon length
N	PEDV-N-F	CTTCTGTCAGYTTTCAGGATCGT	26 378-26 400	59.21	92 bp
	PEDV-N-R	CTTAGAAAGGGGYTTGTCATTAGTA	26 445-26 469	58.88	
	PEDV-N-P	FAM-CGCAAACGGGTGCCATTATCYCT-BHQ1	26 404-26 426	66.33	

Note: The primer sites are based on the complete genomic sequence of the PEDV CV777 strain.（GenBank accession no. AF353511）

Table S1.2 System configuration

Component	Volume（μL）
Forward primer（10μmol/L）	0.4
Reverse primer（10μmol/L）	0.4
Probe（10μmol/L）	0.2
U + One Step RT-qPCR Probe 5 × Master Mix	4
RNA	2
RNase free dd H_2O	13
Total volume	20
