# Supplementary material for: In Vitro Assessment of Antiviral Activity of Saikosaponin B2 Against Porcine Epidemic Diarrheal Virus
Source: Viruses. 2026 Sep 10;18(9):997. doi: 10.3390/v18090997 (PMC13612102; doi:10.3390/v18090997)
Supplement: Supplementary file 1 [file viruses-18-00997-s001.zip › Supplementary Table S2.pdf]

Supplementary Table S2 Cytotoxicity to Vero E6 cells

Name	CC_{50}(umol/L)	CC_{20}(umol/L)	CPE inhibition（%）
Acetylcysteine	87.96	31.99	-0.29 ± 2.64
Naringenin	276.60	252.60	93.43 ± 18.39
Indomethacin	251.10	243.18	7.76 ± 2.20
Molnupiravir	＞512	＞512	99.84 ± 17.62
Baicalein	＞512	＞512	83.18 ± 8.46
Chlorogenic acid	＞400	＞400	1.07 ± 5.65
Andrographolide	168.30	＞512	103.38 ± 18.10
Arctigenin	＞512	＞512	94.98 ± 10.26
Nitazoxanide	371.80	338.34	128.40 ± 7.67
Geniposide	＞400	＞400	37.21 ± 7.66
Betulinic acid	247.40	234.98	86.89 ± 7.02
Aloperine	307.70	257.75	82.92 ± 5.90
Kaempferide	＞400	＞400	10.31 ± 12.58
Saikosaponin B2	228.40	199.49	123.24 ± 17.48
Vidarabine	146.00	88.20	-6.79 ± 4.02
Spermine	＞512	＞512	104.82 ± 7.67
Isoliquiritigenin	61.67	39.89	152.68 ± 17.08
Ginsenoside Rb2	＞400	＞400	7.46 ± 13.54
Limonin	＞512	＞512	73.70 ± 8.14
Gramine	＞400	＞400	16.51 ± 15.02
Phillyrin	＞400	＞400	22.11 ± 20.23
Isoscopoletin	＞400	＞400	24.03 ± 10.12
Camphor	＞400	＞400	26.19 ± 11.29
L-Lysine	＞400	＞400	27.40 ± 7.31
Hederasaponin B	＞400	＞400	13.31 ± 7.23
L-Lysine hydrochloride	＞400	＞400	15.25 ± 5.96
Coumarin	＞400	＞400	26.38 ± 6.68
Cynarin	99.70	55.36	29.95 ± 33.57
Epigoitrin	＞512	＞512	16.59 ± 3.40
Picroside II	257.80	149.69	10.09 ± 6.20
Isoborneol	567.90	＞400	8.41 ± 2.14
Desaminotyrosine	＞400	＞400	9.04 ± 1.67
Curcumin	73.14	62.62	65.51 ± 6.89
Emodin	83.85	32.51	130.83 ± 10.81
1-Docosanol	163.90	15.79	13.87 ± 5.03

Cynaroside	85.71	27.83	35.66 ± 3.58
Shikonin	4.11	3.83	17.26 ± 3.12
Dihydromyricetin	87.73	50.78	7.13 ± 4.25
Brefeldin A	0.85	0.52	-0.48 ± 1.34
Glycitin	116.20	87.22	37.27 ± 4.48
Eriodictyol	46.58	25.68	21.47 ± 6.25
Hyperoside	＞512	＞512	37.13 ± 6.58
Xanthohumol	16.03	14.83	87.39 ± 7.37
Oxyresveratrol	85.72	53.86	26.36 ± 6.10
Betulonic acid	68.57	40.19	100.15 ± 12.36
